# Single Coil Mechano‐Electromagnetic System for the Automatic 1‐Axis Position Feedback 3D Locomotion Control of Magnetic Robots and Their Selective Manipulation

**DOI:** 10.1002/advs.202201968

**Published:** 2022-06-16

**Authors:** Armando Ramos‐Sebastian, Seungchan Hwang, Sung Hoon Kim

**Affiliations:** ^1^ Department of Electronics Convergence Engineering Wonkwang University Iksan 54538 Republic of Korea; ^2^ Wonkwang Institute of Materials Science and Technology Wonkwang University Iksan 54538 Republic of Korea; ^3^ Department of IT Convergence Mechatronics Engineering Jeonbuk National University Jeonju 54896 Republic of Korea

**Keywords:** electromagnetic systems, feedbackless locomotion controls, magnetic locomotion, magnetic microrobots

## Abstract

3D locomotion of magnetic microrobots requires at least one pair of coils per axis and 3D feedback of the position of the microrobot. This results in voluminous systems with high‐power usage and a small working space, which require complex and expensive controllers. This study presents a single‐coil magneto‐electromagnetic system, comprising a parallel robot and coil, capable of precise 3D locomotion control of magnetic millirobots while requiring only feedback of the vertical position of the millirobot. The coil current creates a 2D magnetic trapping point in the horizontal plane, which depends on the position and orientation of the coil and toward which the millirobot moves, eliminating the need for position feedback at such plane. The vertical position of the millirobot is controlled by varying the coil current while receiving feedback from the vertical position of the millirobot. Feedbackless 2D control and 1‐axis feedback 3D automatic control of magnetic millirobots are experimentally demonstrated, achieving higher speeds and similar position errors when compared to control systems with 3D position feedback. Furthermore, selective control of two millirobots is demonstrated by matching the region of maximum vertical magnetic force and the targeted millirobot, achieving selective levitation and control of such millirobots.

## Graphical abstract

A coil creates a 2D magnetic trap (TP),whose position is changed by the translation and tilting of the coil,controlled by a parallel robot. A magnetic robot is dragged toward the TP,allowing feedbackless locomotion in the *XY* plane. Coil current control with feedbackfrom the vertical position of the robot allows for 3D locomotion with only 1‐axis feedback.

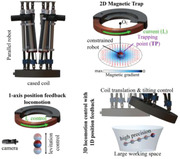

## 1 Introduction

Various studies on magnetic milli/microrobots have focused on their potential biomedical applications such as targeted diagnosis and therapy,^[1–^ ^3^ ^]^ causing the development of their control systems to converge into two different types of stationary electromagnetic systems: 1) those composed of pairs of Helmholtz and Maxwell coils with a large working space that rely on medical imaging for in vivo applications;^[^ ^4^, ^5^ ^]^ and 2) those composed of solenoid coils with microscope cameras for in vitro and superficial in vivo applications owing to their small working space.^[^ ^6^, ^7^, ^8^ ^]^ However, the aforementioned systems heavily depend on the constant tracking of the position of the magnetic microrobot and lack of automatic control mechanisms owing to their focus on biomedical applications, rendering these systems unsuitable for other types of applications such as micromanufacturing.

With the continuing progress of micro‐ and nano‐technologies, the range of applications for magnetic robots has broadened, as well as the need for control systems that can handle such tasks more efficiently. For instance, simple magnetic levitation systems comprising permanent magnets have been developed for density‐based measurements, labeling, and separation of microparticles.^[9–^ ^11^ ^]^ Moreover, control systems for microassembly and micromanipulation have been developed by coupling permanent magnets to the end effector of different robotic manipulators (e.g., robotic arm and cartesian robot).^[^ ^12^, ^13^ ^]^ However, this type of system employs small permanent magnets because the magnetization of permanent magnets cannot be changed dynamically, resulting in small working volumes. Furthermore, vertical position control is not feasible in air, whereas it is hardly attainable in denser fluids with a density close to that of the microrobot due to the relatively slow movement of the end effector,^[^ ^14^ ^]^ rendering these systems suitable only for surface‐constrained applications.

One of the main advantages of the control systems for the locomotion of surface‐constrained magnetic microrobots is the creation of magnetic traps through the interaction of different physical forces (e.g., magnetic and hydrostatic forces). For example, applying a magnetic force to a magnetic microrobot placed at a liquid meniscus surface causes the microrobot to move to specific locations following the applied magnetic force, retaining such a position as long as the same magnetic force is sustained.^[^ ^15^ ^]^ Moreover, the creation of a non‐uniform magnetic field distribution and position control of its maximum point (trapping point [TP]) through the use of magnetized tips have been used to induce the self‐assembly of magnetic swarms and control their locomotion, as well as the locomotion of single microrobots.^[^ ^16^, ^17^, ^18^ ^]^ The aforementioned mechanism can be used for the 2D position feedbackless locomotion control of microrobots because the microrobot is always dragged toward the TP. However, these systems assume that the magnetic robot is light enough to not break the superficial tension of the fluid and precipitate; this may be why this type of control has not yet been widely exploited.

Although substituting permanent magnets for electromagnets at the end effector of the robotic manipulators could enable levitation control and 2D position feedbackless locomotion, most reported systems of this type were conceived for biomedical applications (e.g., capsule endoscopy). Hence, they cannot produce a TP and require constant feedback of the 3D position of the microrobot for their operation.^[19–^ ^21^ ^]^ Recently, 2D position feedbackless locomotion control of single microrobots and magnetic swarms was achieved through a mechano‐electromagnetic system comprising a three degrees of freedom (DOF) parallel robot that controlled the tilting (TIL) of a coil.^[^ ^22^ ^]^ Presumably, this is one of the only two reported systems capable of such a control mechanism, the other being a recently developed static coil system.^[^ ^23^ ^]^ Furthermore, the system can cause precipitated microrobots to float to the surface of the liquid because of the implementation of an electromagnet rather than a permanent magnet. However, such mechano‐electromagnetic systems have a small working space (circular with a diameter of 40 mm) and cannot perform 3D locomotion or selective locomotion control of the microrobots.

Several strategies for the collective locomotion of magnetic microrobots have been proposed,^[^ ^24^ ^]^ with the TP mechanism being the simplest because of its ability to induce the self‐assembly of magnetic swarms and create a constant pushing force toward the TP by energizing the coil.^[^ ^22^ ^]^ Swarm locomotion control can be useful for microassembly and microtransportation application;^[^ ^12^, ^25^ ^]^ however, thus far, it is only applicable in surface‐constrained applications. For large 3D working environments, selective control of different magnetic millirobots is more suitable. However, although 2D selective control of magnetic microrobots is an easy and widely reported task,^[^ ^26^ ^]^ selective locomotion in 3D is not an easy task and has rarely been reported. In the few reported studies where this has been achieved, it is necessary to use robots with different magnetic^[^ ^27^ ^]^ or geometrical properties.^[^ ^28^ ^]^

In our previous work,^[^ ^22^ ^]^ a 3‐DOF (*X*‐axis and *Y*‐axis rotations, *Z*‐axis translation [TRA]) parallel robot was developed to control the rotation of a coil and feedbackless 2D‐locomotion of magnetic microrobots. However, the working surface was small and access to the working space was limited owing to the parallel robot; therefore, 3D locomotion and selective locomotion of Magnetic millirobots (mag‐bots) was not possible. In the present study, a single‐coil mechano‐electromagnetic system, placed above the working volume, consisting of a coil and a six‐DOF (three rotations, three TRA) parallel robot is proposed for the automatic 3D control of magnetic milli/microrobots while requiring only 1‐axis (*Z*‐axis) feedback of the vertical position of the microrobot. Compared to our previous system, the additional DOF and position of the proposed system allow for an additional TP control strategy based on the TRA of the coil, whose main advantages are that it allows 3D locomotion of mag‐bots, selective control of magnetic microrobots, and increases the working volume. In particular, the TIL and translational motions of the coil provide the scalability of the working space and precise control for practical applications.

Similar to other magnetic levitation systems, the vertical position of the magnetic robot referred to as the mag‐bot is controlled by varying the electric current flowing through the coil. As the mag‐bot levitates, it becomes magnetically trapped in the region of the maximum magnetic field (TP), coinciding with the axis of the electromagnet when it is perpendicular to the *XY* plane. The position of the TP changes according to the position and orientation of the coil with respect to the mag‐bot. However, the mag‐bot is always dragged toward the position of the TP. This allows the control of the position of the mag‐bot in the *XY* plane through the TRA and TIL control of the coil using a parallel robot, without requiring feedback of the position of the mag‐bot. Using the proposed system, 2D position feedbackless automatic locomotion control and 3D 1‐axis position feedback automatic locomotion control of a magnetic robot through a helical trajectory are demonstrated, achieving higher speeds and position errors similar to those of control systems with full 3‐axis feedback of the position of the mag‐bot. Moreover, the potential use of the system for automatized processes on biological fluids with a large working space is demonstrated through the position feedbackless 2D locomotion control of a mag‐bot through a channel filled with plasma fluid. Furthermore, a control strategy for the selective control of two mag‐bots was proposed and demonstrated by matching the position of the maximum‐vertical magnetic force and a targeted mag‐bot.

## 2 Single Coil Mechano‐Electromagnetic System Concept

**Figure 1:**
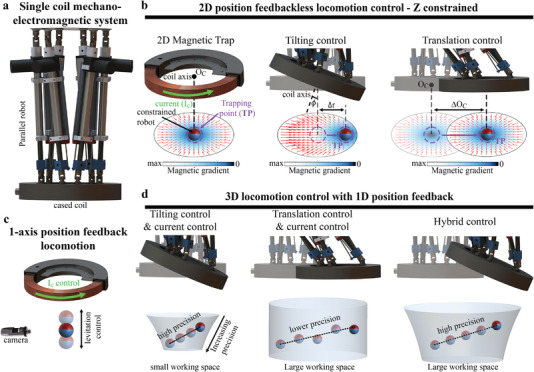
Concept of the single coil mechano‐electromagnetic system for 3D locomotion control using only 1‐axis feedback. a) Mechano‐electromagnetic system. b) Creation of a 2D magnetic trap by a coil and its position control, through the tilting and translation of the coil for 2D position feedbackless locomotion control. c) 1‐axis feedback vertical locomotion through the control of the current flowing in the coil. d) Combination of 2D feedbackless locomotion control mechanisms and 1‐axis position control for the implementation of 3D locomotion control.

The proposed mechano‐electromagnetic system is primarily composed of a coil inside a steel case, which is attached to a six‐DOF parallel robot, as shown in **Figure** **1**a. Considering a circular coil with its origin at O_C_ for any plane perpendicular to the axis of the coil and located further than the critical distance (*d*_c_),^[^ ^22^ ^]^ a gradient magnetic field distribution (*G_r_*) is created with its minimum coinciding with the axis of the coil when an electric current (*I*_c_) flows through the coil. A magnetized object located in a plane with such gradient distribution will be dragged toward the region where the gradient is minimum and remain “trapped” at that location, which is called the TP. As depicted in Figure 1b, TIL the coil by an angle *ϕ* will change *G_r_*, resulting in a displacement of the TP (Δ*r*) with respect to the axis of the coil and causing a mag‐bot to move toward the new location of the TP. Although the TRA of the coil (ΔO_C_) does not alter *G_r_* with respect to the coil, the TRA changes the position of O_C_ and TP with respect to the mag‐bot, causing the mag‐bot to move to match the TP. The locomotion control within this plane can be achieved without any feedback on the position of the mag‐bot because the mag‐bot is always dragged toward the TP and its position depends only on the orientation and position of the coil.

The vertical control of the mag‐bot is achieved through a closed‐loop (CL) control system by varying the magnitude of *I*_c_ while receiving feedback from the *Z*‐coordinate of the position of the mag‐bot, and hence 1‐axis position feedback control, as depicted in Figure 1c. Moreover, the 3D locomotion control of a magnetic microrobot is achieved by merging the 2D position feedbackless locomotion mechanisms and the 1‐axis position feedback levitation control. As shown in Figure 1d, using TIL and current control leads to a conical frustum‐shaped small working space with high‐precision locomotion, whereas employing TRA and current control results in a larger cylindrical working space but with lower precision. A large working space with high‐precision locomotion was obtained by combining the three control mechanisms (hybrid control).

### 2.1 Working Principle

#### 2.1.1 Levitation Control

**Figure 2:**
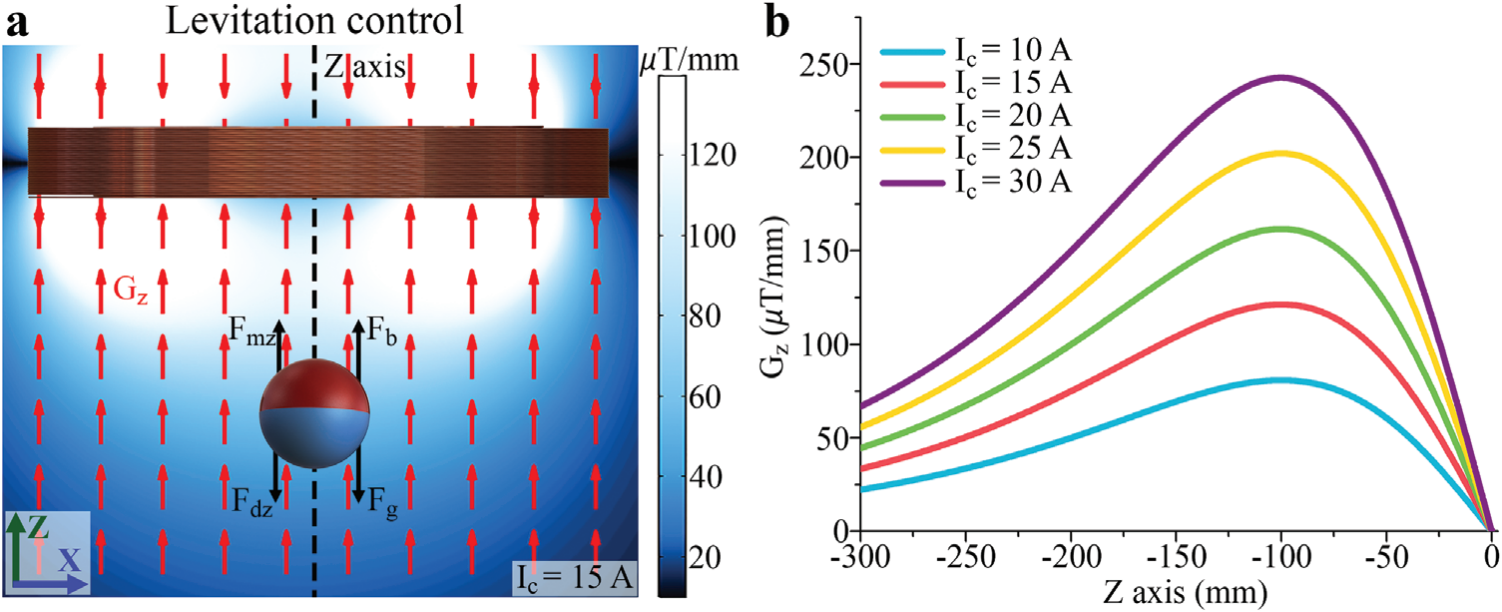
Levitation control. a) Forces acting in a mag‐bot during levitation control and gradient distribution *G_z_* produced by the coil of the system. b) *G_z_* along the axis of the coil for different values of *I*_c_.

During levitation control, the mag‐bot experiences the gravitational force *F*_g_ = *m*_r_ *g* (where *m*_r_ is the mass of the mag‐bot), buoyancy force *F*_b_ = *V*_r_ *ρ*_f_ *g* (where *V*_r_ is the volume of the mag‐bot and *ρ*_f_ is the density of the fluid where the mag‐bot is levitating) that pushes the mag‐bot upwards, *Z* component of the drag force *F*_d_ *_z_* = 6*πμ*_f_ *r*_m_ *v*_f_ *_z_* (where *μ*_f_ is the dynamic viscosity of the fluid, *r*_m_ is the radius of the mag‐bot, and *v*_f_ *_z_* is the *Z* component of the velocity of the mag‐bot in the fluid) that opposes the movement of the mag‐bot, and magnetic force *F*_m_ *_z_* (*Z* component of the magnetic force) expressed in Equation (1), as illustrated in **Figure** **2**a.

Figure 2a illustrates the gradient magnetic field distribution produced by a coil with 600 turns and a radius of 200 mm, which are the same parameters as the coil used in our system. Its axis and origin coincide with those of the *Z*‐axis, with an applied *I*_c_ of 15 A. *F*_m_ *_z_* is given by

(1) $$F_{mz}=G_{z}m$$

where *m* is the magnitude of the magnetic moment of the mag‐bot, and *G_z_* (dot product of the gradient of the *Z*‐component of the magnetic field and the orientation of the mag‐bot) is expressed as

(2) $$G_{z}=\left(a\cdot \nabla B_{z}\right)$$

where *a* is a unit vector that represents the orientation of the mag‐bot. At equilibrium during levitation,

(3) $$F_{mz}-F_{dz}-F_{g}+F_{b}=0$$

As shown in Figure 2b, *G_z_* is not uniform and varies with the position of the mag‐bot. Therefore, the value of *G_z_* must be actively controlled by varying *I*_c_ Therefore, for the mag‐bot to maintain its position during levitation, the position feedback of the vertical position of the robot is required.

#### 2.1.2 2D TP Control Mechanism

2D control of mag‐bots constrained to a liquid surface is required for several applications. In such cases, most systems rely on the surface tension of the liquid to prevent mag‐bots from precipitating, and if they precipitate, these systems lack a mechanism to set the mag‐bots afloat. For our system, the mag‐bot can easily be set afloat to the liquid–air or liquid–liquid interface by increasing the magnitude of *G_z_* that satisfies the condition

(4) $$\left|F_{g}-F_{b1}\right|<F_{mz}<\left|F_{g}-F_{b2}\right|$$

The magnetic force must be greater than the difference between *F*_g_ and the buoyancy force due to the denser fluid (*F*_b1_) but lower than the difference between *F*_g_ and the buoyancy force due to the denser fluid (*F*_b2_). The greater the difference between *F*_b1_ and *F*_b2_, the greater is the range of *G_z_* that allows the mag‐bot to remain at the liquid interface. For the position of the interface with respect to the coil, it is possible to set the mag‐bot afloat without the need for any feedback by knowing beforehand, as demonstrated in a previous study.^[^ ^22^ ^]^

Because of the circular shape of the coil, the gradient magnetic field distribution at any plane perpendicular to the *Z*‐axis depends on the radial distance (*r*) between the axis of the coil and the mag‐bot. Therefore, it is simpler to use cylindrical coordinates to analyze the mag‐bot locomotion. For a surface‐constrained mag‐bot located below the distance from the center of the coil within which the TP is not unique (*d*_c_) (in our system, *d*_c_ = 140 mm), it will be horizontally dragged toward the location of the minimum gradient distribution by the horizontal magnetic force *F*_mr_ (radial component of the magnetic force) while experiencing an opposing drag force *F*_dr_ (radial component of the drag force). *F*_mr_ is expressed as

(5) $$F_{mr}=G_{r}m$$

where *G_r_* is given by

(6) $$G_{r}=G_{x}+G_{y}=\left(a\cdot \nabla B_{x}\right)+\left(a\cdot \nabla B_{y}\right)$$

**Figure 3:**
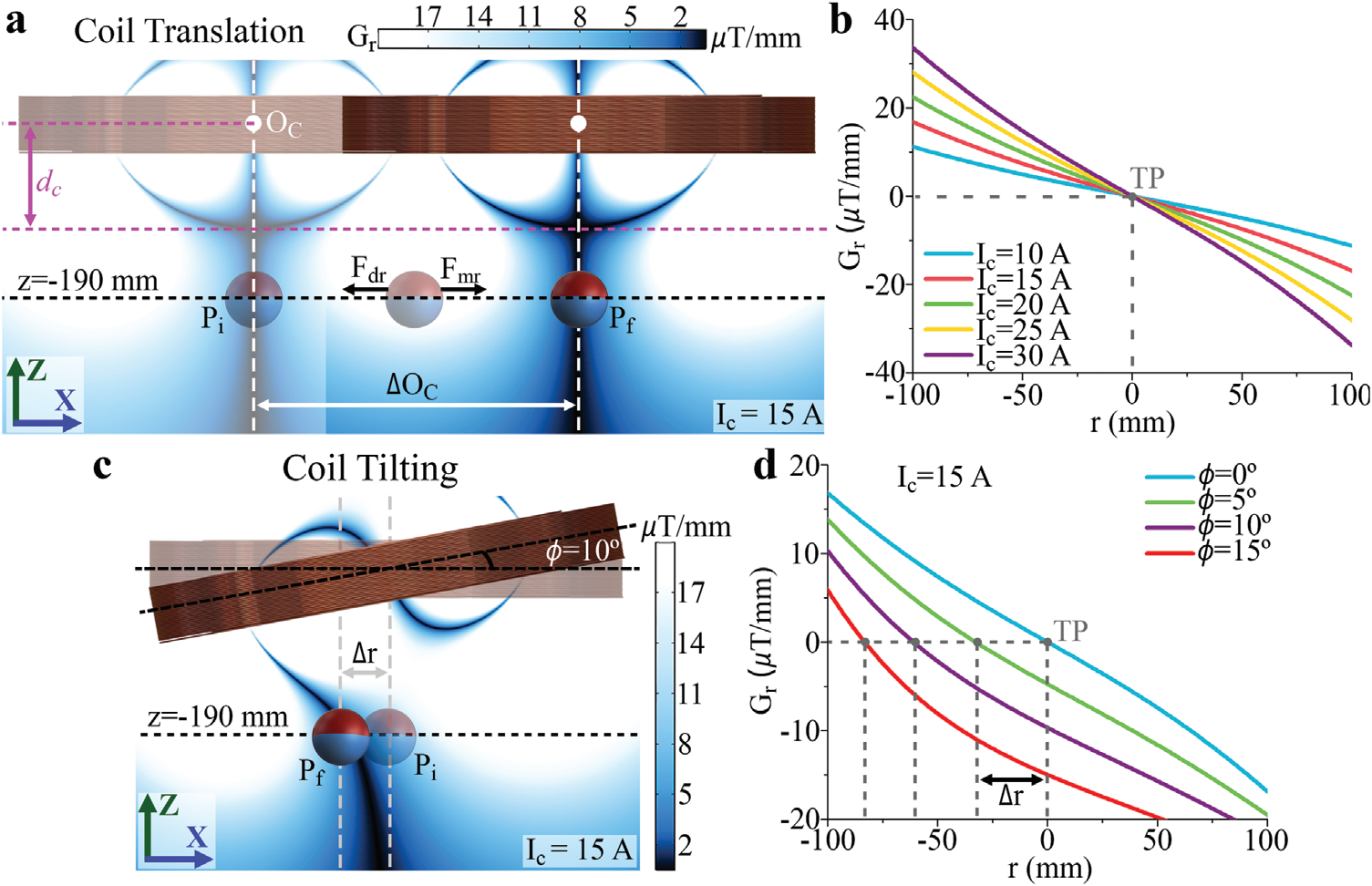
Horizontal position control. a) Change in the gradient magnetic field distribution *G_r_* due to the translation of the coil. b) *G_r_* distribution along the axis *r* for different values of electric current (*I*_c_). c) Change in the *G_r_* distribution due to the tilting of the coil. d) *G_r_* distribution along the axis *r* for different coil tilting angles (*ϕ*).

**Figure** **3**a shows the distribution of *G_r_* at the *ZX* plane produced by our coil when *I*_c_ = 15 A, in which it will be the same for any plane with vertical axis *Z* because of coil symmetry. For separation distances from the axis of the coil further than *d*_c_, there is a single TP at every plane *XY* that coincides with O_C_ for any plane below *d*_c_. As observed, if the coil is translated by an arbitrary distance ΔO_C_ while a mag‐bot is trapped at the TP (*P*_i_, in Figure 3A), the gradient distribution will also be translated by the same distance, dragging the mag‐bot along with it (to *P*_f_, in Figure 3A). Moreover, Figure 3b displays the position of the TP (coordinate *r* = 0 mm) and *G_r_* for different values of *I*_c_ (ranging from 10 to 30 A), showing that the magnetic force dragging the mag‐bot toward the TP increases with the distance between them and with the magnitude of *I*_c_.

The TIL of the coil changes the distribution of *G_r_* compared to the TRA of the coil that does not change the *G_r_* with respect to the coil, as observed in Figure 3c. This results in the TP no longer coinciding with O_C_, but instead being displaced by a distance Δr, which increases as the separation distance with the coil decreases. This can be observed through the black line touching the coil at *z* = 0, which converges toward the *Z*‐axis as the distance increases. For example, a mag‐bot previously trapped at the TP originally located at *Z* = −190 mm below O_C_ will be horizontally displaced by ≈63 mm when the coil is tilted to *ϕ* = 10°. Figure 3d shows the change in the distribution of *G_r_* when different angles tilt the coil, demonstrating that *r* also increases as *ϕ* increases.

Therefore, the horizontal position of a mag‐bot can be controlled without requiring any feedback of its position, just through the TRA and rotation of the coil to control the position of the TP.

### 2.2 Parallel Robot

**Figure 4:**
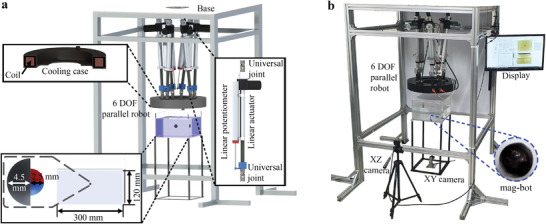
a) Schematic and b) photograph of the experimental setting consisting of the single‐coil mechano‐electromagnetic system, a mag‐bot, and a container filled with a glycerin–water mix.

The TRA and TIL of the coil were controlled by a six‐DOF parallel robot, as shown in **Figure** **4**. The parallel robot steel base was suspended 1.6 m above the ground using 40 mm × 40 mm aluminum profile. The bases of the six linear actuators (Motorbank, LM‐Z17, Korea) were fixed equidistantly to the steel base through universal joints. The tips of the moving rods were fixed to a cylindrical container with universal joints. A coil with coil with 600 turns and a radius of 200 mm was made using an EI/AIW enamel wire with a diameter of 2 mm; the coil had an inductance of 220 mH and a resistance of 3.7 ohm. The coil was placed inside the container where water flowed to cool it down and was separated 73.5 cm from the base. Both ends of each linear actuator were coupled to linear potentiometers (OPKON ERTL‐300‐D14) to measure the length of the linear actuators.

For the control of the parallel manipulator a graphical user interface (GUI) was designed using LabVIEW. The control program mainly consists of an image display and tracking module, TP position control module, parallel robot position control module, and coil current control module; all developed using mainly LabVIEW. The user can choose in the GUI different TP position control modes, that is, a combination of TRA locomotion, rotation locomotion, feedbackless or full position feedback control, and hybrid mode. The electrical hardware and control equations of the parallel robot are detailed in Section S1, Supporting Information.

Unless otherwise specified, for the experiments, a PLA 3D printed mag‐bot (mag‐bot 1 [MB_1_]) with a radius of 4.5 mm containing a spherical neodymium magnet with a radius of 2.5 mm was used. The mag‐bot was immersed in a 300 mm × 300 mm × 120 mm container filled with a glycerin–water mixture with an *μ*_f_ of 45.38 mPa s. The maximum separation distance between the coil and robot at which control is possible depends on the properties of the mag‐bot; for MB_1_ it is ≈275 mm (see Section S2, Supporting Information).

## 3 2D Position Feedbackless Locomotion Control

To control the locomotion of the mag‐bot in the *XY* plane, the desired set of positions for the TP (toward which the mag‐bot will be dragged) is input in cylindrical coordinates, where *r*_ref_ (reference input for r) and *θ*_ref_ (reference input for angular coordinate [*θ*]) are the desired radial distance and angular coordinate, respectively. TRA locomotion control uses a constant TIL of the coil, *ϕ* = 0°, and O_C_ is displaced to match the desired position of the TP, whereas for rotation control, *ϕ* is modified according to the desired position of the TP.

Although one of the objectives of this study is to develop a 2D position feedbackless locomotion control system, CL control systems were also implemented to compare their performances. Four 2D locomotion controllers were designed using the feedbackless (open‐loop [OL]) and CL locomotion control of a mag‐bot using TIL or TRA control of the coil. During feedbackless locomotion control, the system cannot determine the time the mag‐bot has reached the desired position; therefore, a time parameter Δ*t* needs to be set by the user. The system changes the current position of the TP to the next after the time Δ*t* has elapsed. In this case Δ*t* (1 s) was experimentally found by varying it while making the microrobot follow a straight line. The detailed block diagrams, step responses, and control equations for the four controllers are detailed in Section S3, Supporting Information.

**Figure 5:**
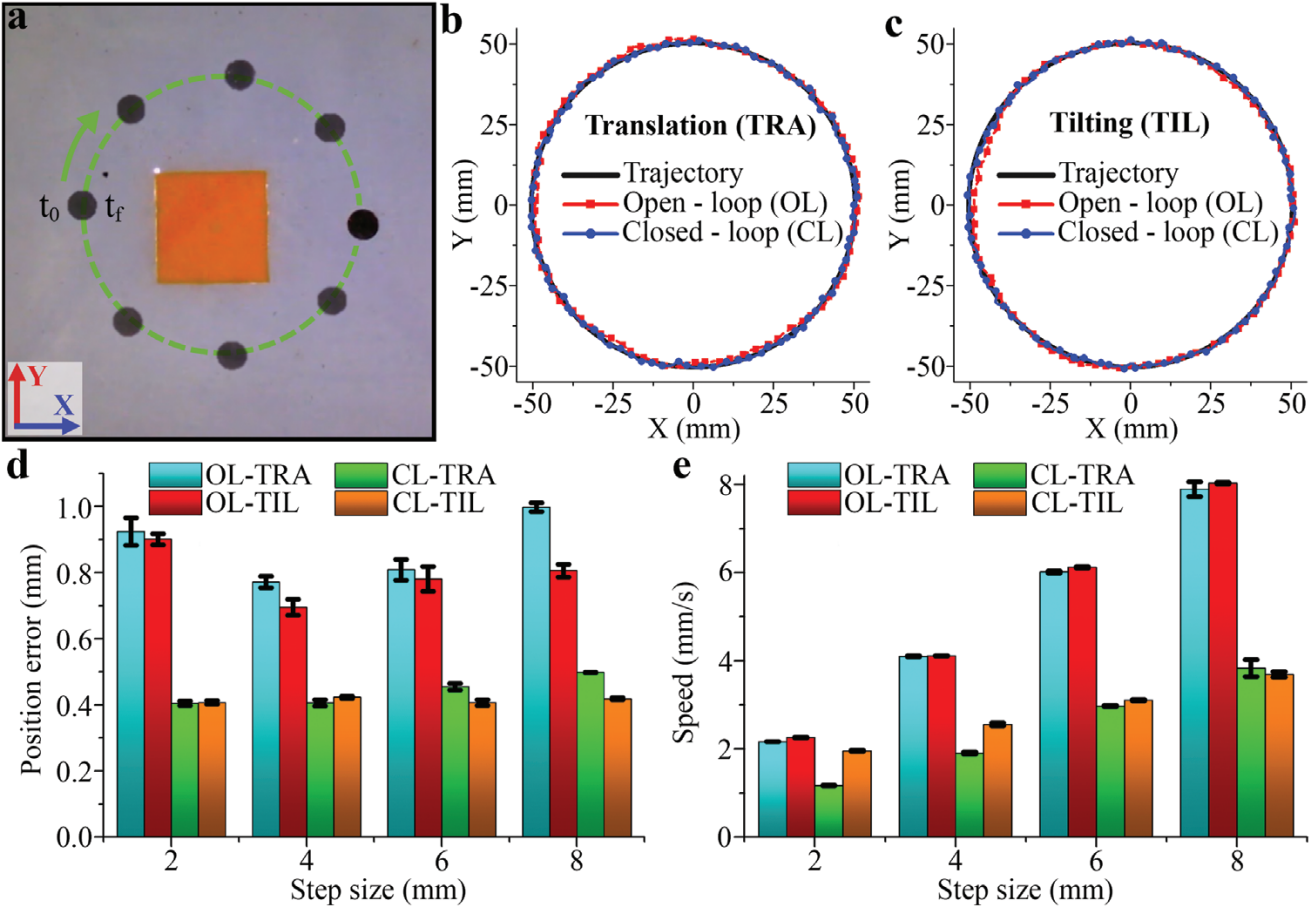
a) Timelapse images of the mag‐bot along a circular trajectory. Graphs comparing the open‐loop (OL) and closed‐loop (CL) 2D locomotion control of the mag‐bot using b) translation (TRA) and c) tilting (TIL) control of the coil. d) Position error and e) speed comparison graphs between the four 2D locomotion methods.

For the four 2D locomotion control systems, the locomotion of the mag‐bot along a circular path with a radius of 50 mm was performed to test the locomotion performance shown in **Figure** **5**a. For this purpose, the circular trajectory was divided into equidistant points of 2, 4, 6, and 8 mm to analyze the effect of different step sizes on the locomotion of the mag‐bot. Figures 5b and 5c show the measured position of the mag‐bot during its locomotion for the TRA and TIL systems, respectively. As expected, the measured positions for both CL‐TRA and CL‐TIL perfectly match the desired trajectory, whereas slight mismatches can be observed for OL‐TRA and OL‐TIL. The measured mean position error, speed for each control system, and the step size are shown in Figures 5d and 5e, respectively. As observed, the speed increased for all systems with higher step sizes, in which the OL systems on average are twice as fast as the CL systems. The speeds of the OL‐TRA and OL‐TIL were similar, which were ≈8, 6, 4, and 2 mm s^−1^ for step sizes of 2, 4, 6, and 8 mm, respectively. On the other hand, the speed for the CL‐TRA was significantly lower than that of the CL‐TIL system for step sizes of 2 and 4 mm, indicating that the system takes a longer time to compensate for the position errors during TRA motions. The position error for both OL systems was higher than that for the CL systems. For respective step sizes of 8, 6, and 4 mm, the position error of both OL systems decreased with the step size, that is, 0.99, 0.81, and 0.77 mm for OL‐TRA and 0.81, 0.78, and 0.69 mm for OL‐TIL, respectively. However, the error slightly increased when the step size decreased to 2 mm, which might indicate that the magnetic force produced during the experimental conditions was weak and could not push the mag‐bot toward the TP.

## 4 3D Locomotion Control 1‐Axis Position Feedback

It is possible to attain 3D locomotion control of a mag‐bot while only requiring feedback of the vertical position of the microrobot by combining the control systems implemented in the previous section with levitation control. To control the vertical position of the mag‐bot, the desired vertical position *Z*_ref_ (reference input for *Z*) is fed to the system and compared with the measured position of the robot (performed by the *XZ* camera) to increase or decrease the value of *I*_C_ using a PID controller. The four 3D controllers are the same as their 2D versions, but with the addition of levitation control. The detail block diagrams, step responses, and control equations for the four controllers are detailed in Section S4, Supporting Information.

**Figure 6:**
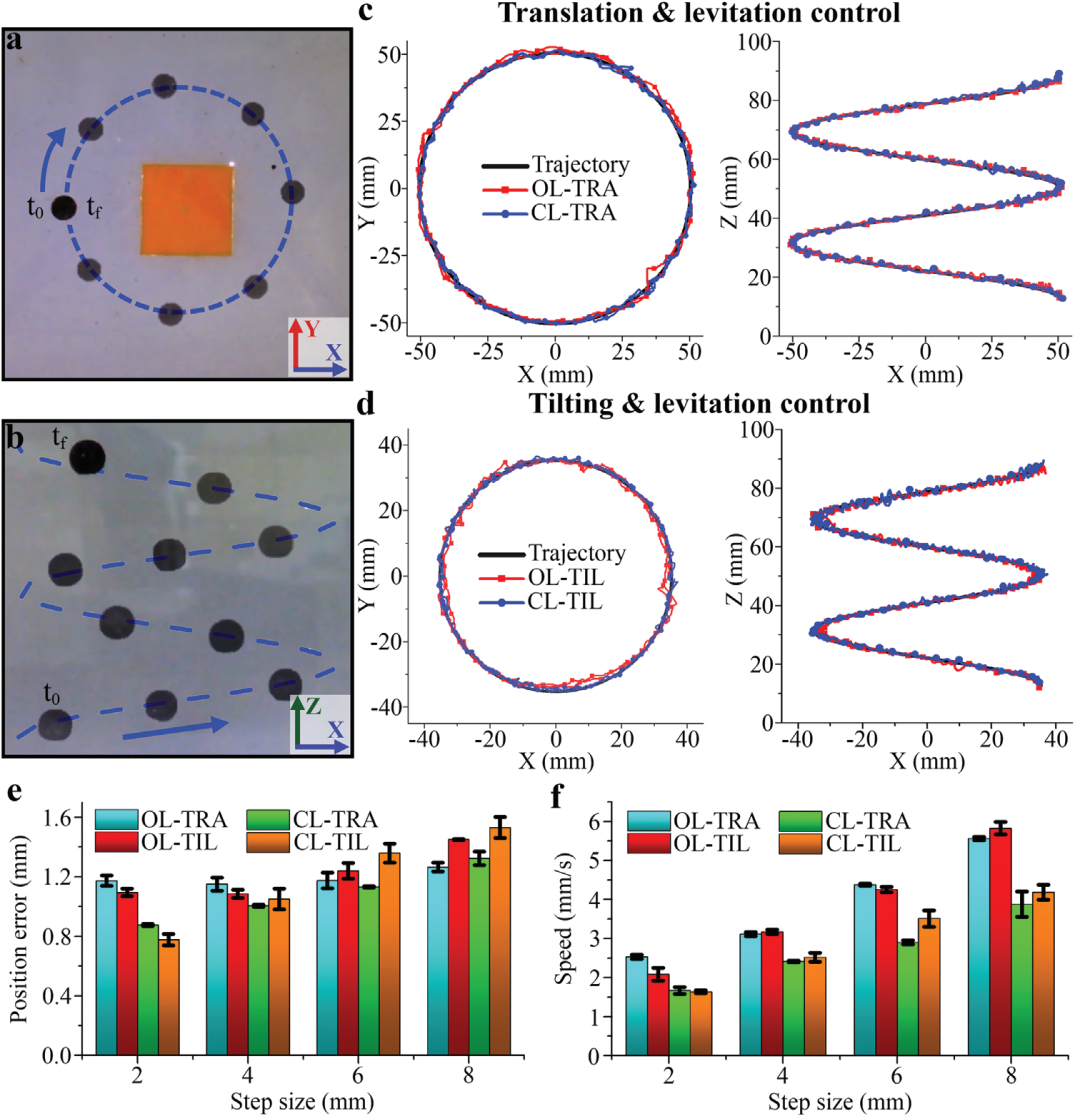
Timelapse images in the a) *XY* and b) *ZX* plane of the mag‐bot following a 3D helical trajectory. Graphs comparing the open‐loop (OL) and closed‐loop (CL) 3D locomotion control of a magnetic microrobot combining levitation control with c) translation (TR) and d) rotation (RT) control of the coil. e) Position error and f) speed comparison graphs between the four 3D locomotion methods.

To analyze the performance of the four 3D control systems, the locomotion of the mag‐bot was tested along a 3D helicoidal trajectory (see Videos S1 [TIL control] and S2 [TRA control], Supporting Information). The radii of the trajectory for the TRA and TIL control systems were 50 and 40 mm, respectively. Timelapse images of the locomotion of the mag‐bot along the helical trajectory in the *XY* and *ZX* planes are shown in **Figures** **6**a and 6b, respectively. The measured positions in the *XY* and *ZX* planes of the mag‐bot following the helicoidal trajectory for the TRA and TIL control systems are shown in Figure 6c,d. The graphs of the *XY* planes show that the position of the mag‐bot for the CL systems matches the desired trajectory, whereas for the OL systems, there are some deviations from the input trajectory. The graphs for the *XZ* planes showed no significant differences between the control systems. The mean position error and speed of the mag‐bot for each of the four control systems are shown in Figure 6e,f. The accuracy of the CL systems was not significantly lower than that of the OL systems, except for a step size of 2 mm.

For this step size, the mean errors of the CL‐TRA and CL‐TIL were 0.87 and 0.77 mm, respectively, whereas for the OL‐TRA and OL‐TIL, they were 1.17 and 1.09 mm, respectively. Furthermore, the accuracy of the CL systems was worse than that of the OL systems for a step size of 8 mm. Overall, the OL systems achieved a faster locomotion speed than the CL systems, that is, 5.56, 5.82, 3.88, and 4.18 mm s^−1^ for OL‐TRA, OL‐TIL, CL‐TRA, and CL‐TIL systems, respectively. For the OL‐TRA, OL‐TIL, CL‐TRA, and CL‐TIL systems, the locomotion speed decreased 2.53, 2.08, 1.67, and 1.63 mm s^–1^ as the step size decreased, respectively.

## 5 System Applications Using Hybrid Mode

The TIL control offers faster and more precise locomotion when compared to TRA control based on the discussions in previous sections. However, its range is small, particularly as the distance between the coil and the mag‐bot increases. Despite the lower speed and precision of the TRA control, its working range is large. Therefore, the optimal control mechanism combines both TIL and TRA mechanisms to obtain a hybrid control system with high precision and a large working space.

The hybrid control system uses TIL motion for locomotion in complex trajectories, while the TRA motion is used only to displace the O_C_ to expand the range of the TIL control or for locomotion in straight lines. Furthermore, no feedback from the *XY* coordinates of the mag‐bot is fed to the control system.

**Figure 7:**
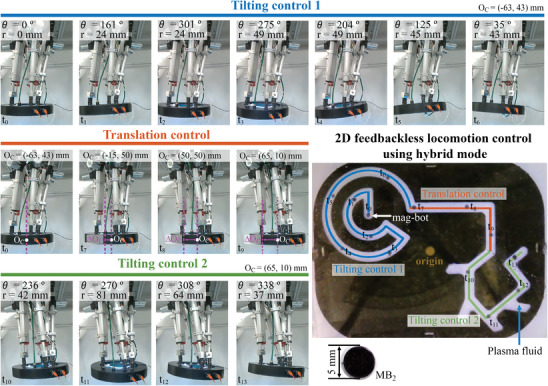
Timelapse images of the parallel robot, coil case, and the 2D feedbackless locomotion control of a mag‐bot along a maze using hybrid control (translation and tilting) for a precise and large working space.

To test this hybrid control system, 2D feedbackless automatic locomotion control of a mag‐bot (mag‐bot 2 [MB_2_]) within a plasma solution (*μ*_f_ of 1.16 mPa s) filled 2D maze with a working space of 220 mm × 180 mm was tested, as shown in **Figure** **7** (see Video S3, Supporting Information). Figure 7 shows timelapse images of the locomotion of MB_2_ along the maze as well as the position of the coil. The maze comprised three different trajectories: a trajectory composed of arcs connected by straight lines with a circular working space of radius 52 mm (blue line), a trajectory composed of two large straight lines (red line), and a trajectory composed of short straight lines with different angles (green line).

For the first trajectory, TIL control was used with O_C_ = (−63, 43) mm. At starting time for locomotion (t_0_), MB_2_ is located at O_C_, and the control parameters *r* and *θ* are both zero based on the timelapse images. For t_1_ and t_2_, *r* was 24 mm, and only *θ* changed because MB_2_ followed a circular trajectory. Likewise, for t_3_–t_6_, *r* is similar (43–49 mm), whereas *θ* changes according to the desired locomotion direction along the arc. The TRA control was used to displace the O_C_ 128 mm to the right and 23 mm below to settle the coil axis at O_C_ = (65, 10) mm when MB_2_ reached the end of the first trajectory. Timelapse images t_0_, t_7_, and t_8_ of the coil system show that the coil moves to the right, increasing ΔO_C_ and dragging MB_2_ with it. At t_9_, the coil appears larger because it moves in the direction of the camera. Then, the TIL control was used again for the remaining trajectory. In this case, because the points are not equidistant from the O_C_, the values of *r* vary significantly, whereas *θ* only changes in a small range of ≈100° (from 236° to 338°).

An additional functionality of the system is the ability to individually control different mag‐bots aided by the gravitational force. When there is more than one mag‐bot, any targeted mag‐bot can be selectively controlled by producing a magnetic force strong enough to levitate only a mag‐bot located at its center, which is the position where the maximum gradient field is produced (see Section S5, Supporting Information). Using this principle, the individual locomotion of the two mag‐bots was demonstrated.

**Figure 8:**
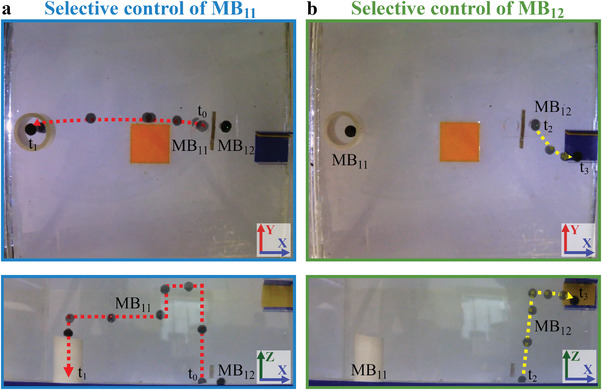
Timelapse images in the *YX* and *ZX* plane of a) the selective locomotion of mag‐bot 1 (MB_11_) toward a pipe, and b) the selective locomotion of mag‐bot 2 (MB_12_) to a container.

**Figure** **8** displays the timelapse images of the selective locomotion of two mag‐bots identical to MB_1_ (MB_11_ and MB_12_) toward different targets (see Video S4, Supporting Information). At the beginning of the experiment (t_0_), both mag‐bots are placed separated by a distance of 15 mm using a piece of acrylic with two holes to prevent them from attracting each other. First, the O_C_ is placed above MB_11_ and the current control system is activated so that *I*_c_ starts increasing until MB_11_ rises and is detected by the control system. *I*_c_ decreases as soon as the control system detects that MB_11_ is levitating; hence, MB_12_ does not levitate. Then, the position of MB_11_ is controlled, moving it over a white pipe and placed within it (t_1_), as shown in Figure 8a. During the time that MB_11_ was moving, MB_12_ remains at its initial position. Then, O_C_ is placed above MB_12_ and the current control system is turned on again (t_2_), resulting in the selective levitation of MB_12_. After that, the position of MB_12_ is controlled, while MB_11_ remains at its position, moving MB_12_ to a small container locater at the right superior corner of the working space, where MB_12_ is placed.

**Figure 9:**
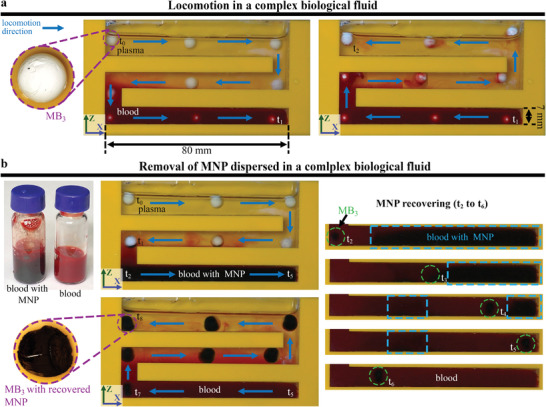
a) Locomotion of mag‐bot 3 (MB_3_) along a maze filled with plasma and blood. b) Retrieval of magnetic nanoparticles dispersed in blood using MB_3_.

To test the viability of the system for the locomotion of mag‐bots in biological complex fluids, the locomotion of a mag‐bot (MB_3_) was tested within an acrylic maze which contained sheep blood, as shown in **Figure** **9**a. Because the mag‐bot cannot be distinguished clearly when it moves in blood, the acrylic maze was filled with sheep blood at the bottom and plasma solution at the top. The locomotion of MB_3_ starts in plasma at the top right corner of the maze (t_0_) and moves toward the lower part of the maze, which contains blood (see Video S5, Part A, Supporting Information). The robot is barely visible (appears as a small white spot) when it moves in blood. After reaching the end of the maze (t_1_), MB_3_ is moved back toward its initial position, located at the superior part of the maze (t_2_). As MB_3_ leaves the section of the maze containing blood, MB_3_ drags along some blood to the plasma‐filled section.

To demonstrate the potential applications of the developed system, the retrieval of magnetic nanoparticles (MNP) dispersed in blood was tested by using the same acrylic maze as in the previous experiment, as shown in Figure 9b (see Video S5, Part B, Supporting Information). Retrieval of MNP that remain in the system after drug delivery and hyperthermia applications is an issue that has been investigated recently by other research groups.^[29,^ ^30^ ^]^ As observed in Figure 9b, the blood containing MNP has a darker, red color appearance. At the beginning of the experiment, MB_3_ is located at the top right corner of the maze (t_0_) and moves from the plasma‐filled toward the blood‐filled area. In the middle of its trajectory, MB_3_ drags some blood that was sedimented in the plasma section, while the MNP in the blood got attached to the lower part of MB_3_ (t_1_). As soon as MB_3_ reaches the blood‐filled section (t_2_), the MNP surrounding the mag‐bot gets attached to it, thus causing the color of the blood to revert to its original state. As MB_3_ continues its locomotion toward the end of the maze (t_5_), changes in the color of the blood in the sections in which MB_3_ passes though can be observed, and the mag‐bot can be distinguished as a black spot (green circle). Areas containing MNP are marked within blue rectangles and exhibit a darker, red color. During its locomotion from t_2_ to t_3_, some MNPs were left behind by MB_3_. However, they were successfully recovered on their way back from t_5_ to t_6_. After MB_3_ retrieved the MNP, it was moved back to its original position at the upper right corner of the maze (t_7_–t_8_). A change in the volume and color of MB_3_ is observed owing to the MNP attached to it. Furthermore, its speed decreased owing to its weight increase.

## 6 Discussion

The system proposed in this study mainly comprises an electromagnet attached to the end effector of a six‐DOF parallel robot. As demonstrated through the experimental results in Section 3, the coil generates a 2D TP that coincides with the axis of the coil toward which the mag‐bot is dragged and constrained. The position of the mag‐bot can be known without position feedback because the mag‐bot moves by itself toward the TP. Hence, 2D automatic feedbackless locomotion control of a mag‐bot was obtained through position control of the TP, controlled by the TRA, and rotation of the coil.

Then, 3D locomotion was achieved by implementing levitation control of the mag‐bot through the control of *I*_c_ while receiving feedback only from the vertical position of the mag‐bot. Although there is no minimum viscosity for which the system cannot be used, the sensor used to measure the *Z*‐axis position of the mag‐bot needs to be significantly fast. In its current state, neither levitation in air (*μ*_f_ = 18.1 *μ*Pa s) nor in plain water (*μ*_f_ = 1 mPa s) is possible in the system owing to the slow frame rate of the camera (30 FPS). Therefore, the 3D locomotion experiments were performed in a glycerin–water mix. To achieve levitation in lower viscosity fluids, a faster camera than the one used in the present study can be used, which will require more computing power. However, a better approach is placing at the center of the coil an array of ultrasonic or electro‐optical sensors, which requires remarkably low computing power and have a significantly fast response time. The performances of both the feedbackless 2D and 1‐axis feedback 3D locomotion control systems (OL systems) were compared to those of full‐position feedback 2D and 3D control systems (CL systems). The experimental results showed that the OL systems were significantly faster than the CL systems, with only a mean position error twice as large for 2D locomotion, but surprisingly not too different in the case of 3D locomotion.

As observed in the results presented in Figures 5e and 6f the locomotion speed of the microrobot can be controlled by the step size of the input trajectory, which has a slight influence on the precision of the system. This is because we experimentally determined a Δ*t* that works similarly for the four step sizes. However, varying Δ*t* is another possible way to control the speed of the mag‐bot. Increasing the value of Δ*t* will result in an average slower locomotion but can improve the resolution, whereas decreasing it will increase the speed of the robot but decrease the precision if Δ*t* is too small. The main factor limiting the value of Δ*t* is *μ*_f_; therefore, for 2D locomotion, the speed and precision can be further increased by applying a higher magnetic force (through the control of *I*_c_). However, this is not the case for 3D locomotion, because *I*_c_ needs to be limited within a certain range in accordance with the vertical position of the mag‐bot. In consequence, it is possible to set *G_r_* strong enough to compensate for a possible change in the flow of the liquid for 2D applications that require it (e.g., microdelivery against a time constant flow); however, 3D locomotion applications are limited to fluids at rest or with a small and constant flow, otherwise 3D feedback of the microrobot is required.

As demonstrated, only a robotic four‐DOF (TRA and TIL along the *X*‐ and *Y*‐axes) end‐effector is required for the 3D locomotion of the mag‐bot using a single electromagnet. This is because the rotation around the coil axis has no effect on the mag‐bot, and TRA along the *Z*‐axis is not necessary because *I*_c_ controls the vertical position of the mag‐bot. However, if available, the TRA along the *Z*‐axis can increase the working space or help maintain a constant vertical distance between the coil and the mag‐bot for a constant resolution while using TIL control.

The calculated positions of the mag‐bot using Biot–Savart law and the measured position of the mag‐bot were quite similar (see Section S3, Supporting Information), demonstrating that the TP can be accurately estimated through calculations. The main reason for the small errors between the calculated and measured values is the existence of some imperfections while manufacturing the system, and the negligible gradient near the TP. However, as observed through curve fitting of the measured data, the system was able to operate with mean errors below 400 and 800 *μ*m for 2D and 3D locomotion, respectively. Considering that each pixel in the camera has a length of ≈280 *μ*m, these measured position errors are close to the maximum resolution that our cameras can resolve. Considering this, the accuracy of the system will greatly improve if cameras with a higher resolution are used for curve fitting. Furthermore, it is possible to increase the precision and speed of the system by implementing deep learning algorithms.

Using hybrid locomotion, the 2D feedbackless automatic locomotion of a mag‐bot in a large maze filled with plasma fluid was successfully demonstrated. For other systems, constant feedback on the position of the robot is required even if the trajectory in which the robot must follow is known beforehand. However, as demonstrated in our system, the robot followed the programmed trajectory without any feedback after the trajectory was input. This demonstrates that the system is suitable for automatization tasks in which the locomotion trajectory is known beforehand. In addition, the locomotion of the mag‐bot in the plasma fluid and blood demonstrated that the system could be used for in vitro applications. Furthermore, the experiment demonstrated the need for both the TRA and TIL control of the coil. Although TIL offers higher precision control, its working space is small. However, the TRA allows an increase in the range of the system.

Selective 3D locomotion control of two identical mag‐bots was demonstrated by matching the position of the targeted mag‐bot with the axis of the coil (location of the maximum magnetic field) to produce levitation of the mag‐bot while the other remained sunk. This selective locomotion mechanism can be further refined by exploiting the densities and magnetization values of different mag‐bots to increase the number of mag‐bots that can be individually controlled. Furthermore, the size of the coil can be reduced to better target the mag‐bots and increase the gradient magnetic field. Moreover, the system is capable of controlling the locomotion of mag‐bots with different shapes (change in dragging force) and magnetic properties (change in magnetic force) as long as Equation (4) is satisfied, which was demonstrated in our previous work.

The system focuses on decreasing the required position feedback from three coordinates to one. In consequence, when operated in the automatic mode, for a successful control, the trajectory and locomotion ambient for the microrobot must be known beforehand, making it suitable for repetitive tasks in well‐controlled and characterized environments, such as manufacturing and transportation. The 1‐axis feedback automatic 3D locomotion mode is not suitable for dynamic environments; hence, its bio‐applications are limited only to in vitro applications. When operating in dynamic environments, manual locomotion is required; therefore, the user can adjust the parameters to compensate for the changes in the environment (e.g., time variable flow and unknown obstacles). This system was not designed for in vivo applications, however, because all the space below the coil is free of any obstruction, it is easy to combine the system with existing medical imaging technology so that the user can steer the mag‐bots in vivo.

## 7 Conclusion

Although the feedbackless 2D locomotion of mag‐bots is feasible using permanent magnets attached to the end effector of robots, such systems are too slow to achieve levitation control and require larger robots when compared to electromagnets. Moreover, there are several configurations of electromagnetic systems that have been developed to control magnetic milli/nanorobots. However, all of them require feedback from the 3D coordinates of the position of the microrobot for locomotion control. Furthermore, the controllers need to actively compensate for several parameters to retain the position of the mag‐bot at a given point, increasing the processing time, complexity of the algorithms, hardware requirements, and costs. In a previous study, we developed a 2D position feedbackless locomotion control system using a TP controlled by a three‐DOF parallel robot and a coil. However, its main drawbacks were that the working surface was small, selective locomotion of mag‐bots was not possible, access to the working space was hindered by the position of the parallel robot, and the resolution of the system changes with the distance between the mag‐bot and the coil.

Consequently, we propose a single‐coil mechano‐electromagnetic system for the automatic 3D locomotion of mag‐bots, while requiring only feedback of the vertical position of the mag‐bot. The system mainly comprises a six‐DOF parallel robot and a coil. Control of the electric current flowing through the coil while receiving feedback of only the vertical position of the mag‐bot resulted in levitation control, which in combination with the 2D TP allowed the control of 3D position of the mag‐bot with only 1‐axis feedback. The additional DOF of the parallel robot resulted in an additional TP control mechanism through the TRA of the coil, which also yielded a high working volume. Moreover, even if the mag‐bot moves in the vertical axis, the distance between the coil and the mag‐bot can be maintained constant, and thus, the resolution of the TP control through the rotation mechanism. Furthermore, the change in the position of maximum magnetic gradient produced by the TRA of the coil allowed to selectively control different mag‐bots by positioning the maximum magnetic gradient over the target mag‐bot.

In our future work, we will focus on the implementation of artificial intelligence algorithms to increase the precision and locomotion speed of mag‐bots using this system and the substitution of the camera for a faster sensor suitable for levitation in air. Furthermore, we plan on working on 3D locomotion control of magnetic microswarms.

## 8 Experimental Section

### Fabrication of Mag‐Bots

The shell of MB_1_ was fabricated using a black PLA filament and 3D printer (Ultimaker S3). The shell consisted of two hollowed semispheres with external and internal radii of 4.5 and 2.5 mm, respectively. An N35 ball magnet, which had a radius of 2.5 mm, was placed between the semispheres to bond the three components. MB_2_ was an N35 disk magnet with a radius of 2.5 mm and height of 1 mm. It was painted black using a permanent marker to improve the visibility. MB_3_ was an N35 ball magnet with a radius of 2.5 mm. It was painted white using spray paint.

### Preparation and Characterization of Fluids

The fluid used for most locomotion experiments was prepared by stirring seven parts of glycerin with three parts of tap water. On the other hand, a commercial plasma solution (HK inno.N, Korea) was used as the fluid of the locomotion experiment of MB_2_. The kinematic viscosities of both the fluids were measured using a digital viscometer (Brookfield DV1MLVTJ0). The blood used in the experiments was sheep blood (Biozoa Biological Supply, Seoul, Korea). In the experiment conducted to retrieve MNP dispersed in blood, iron oxide MNPs with average sizes in the range of 80–100 nm (Fe_3_O_4_‐100, GetNanoMaterials, Oocap Inc., Las Cruces, USA) were added to sheep blood at a concentration of 11 mg mL^−1^ and stirred mechanically.

### Magnetic Field Simulations

The magnetic field of the coil was calculated using Biot–Savart's law, and the position of the TP was calculated by calculating the magnetic gradient assuming that the mag‐bot instantly aligns with the direction of the magnetic field. The calculations were performed in MATLAB (MathWorks, USA).

### Position Measurement

Two 30 FPS USB cameras with a resolution of 1280 × 960 pixels (WITHROBOT, oCam‐5CRO‐U‐M) were used to measure the position of the microrobot using the vision and motion module of LabVIEW (National Instruments, USA). One camera was placed below the container to measure the position of the robot in the *XY* plane and another to measure the position in the *ZX* plane.

### Video Editing

For the elaboration of the videos in Supporting Information, the LabVIEW GUI (displaying the *XY* and *ZY* plane cameras) and the coil system (displayed using an additional USB webcam [APC930U, ABKO] and the Windows Camera application) screens were recorded using oCAM (ohsoft) while performing the respective locomotion tests. Subsequently, the required parts of the recorded screen were trimmed and processed using Adobe Premiere Pro (Adobe, USA).

### Control Program

The main control program was a GUI, which was developed using LabVIEW. The GUI was used for current sensing, inputs of the control parameters, 3D trajectory, and video recording (see Figure S2, Supporting Information).

### Statistical Analysis

All experiments were performed at least three times, with similar results. Results in all graphs were presented as mean values ± standard error of the mean.

## Conflict of Interest

The authors declare no conflict of interest.

## Supporting information

Supporting Information

Supplemental Video 1

Supplemental Video 2

Supplemental Video 3

Supplemental Video 4

Supplemental Video 5

## Data Availability

The data that support the findings of this study are available from the corresponding author upon reasonable request.
