# Supplementary material for: Single Coil Mechano‐Electromagnetic System for the Automatic 1‐Axis Position Feedback 3D Locomotion Control of Magnetic Robots and Their Selective Manipulation
Source: Adv Sci (Weinh). 2022 Jun 16;9(23):2201968. doi: 10.1002/advs.202201968 (PMC9376823; doi:10.1002/advs.202201968)
Supplement: Supplementary file 1 — Supporting Information [file ADVS-9-2201968-s003.pdf]

ADVANCED SCIENCE
Open Access

Supporting Information

for *Adv. Sci.*, DOI 10.1002/advs.202201968

Single Coil Mechano-Electromagnetic System for the Automatic 1-Axis Position Feedback 3D Locomotion Control of Magnetic Robots and Their Selective Manipulation

*Armando Ramos-Sebastian, Seungchan Hwang and Sung Hoon Kim**

Supporting Information

Single Coil Mechano-electromagnetic System for the Automatic 1-axis Position Feedback 3D Locomotion Control of Magnetic Robots and their Selective Manipulation

*Armando Ramos-Sebastian, Seungchan Hwang, and Sung Hoon Kim**

Section S1. Parallel Robot Hardware Configuration

The system uses the main PC to control the position of the parallel robot and locomotion of the mag-bot, as shown in **Figure S1**. Two USB cameras, a current sensor (WCS6800, Winson), and linear potentiometers were connected to the computer to monitor the mag-bot position (P_r), I_c, and the linear actuator length. Based on the user input parameters and feedback from the sensors through a DAQ (NI USB-6008), the main computer calculated the required I_c for levitation control and the output length of each linear actuator for the translation and tilting control of the coil. Moreover, I_c is controlled by PWM control using an IGBT (FZ900R12KE4, Infineon), a MCU (Arduino Due), and a 120 V, 3 kW SMPS (CSP-3000-120), while the linear actuators are controlled by motor drivers (BTS-7960) and a 24 V, 600 W SMPS (SE-600-24).

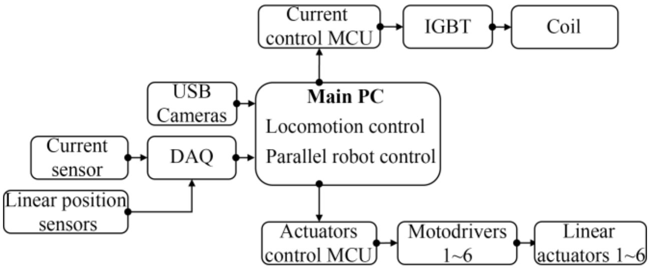

Figure S1. Hardware comprising the mechano-electromagnetic system.

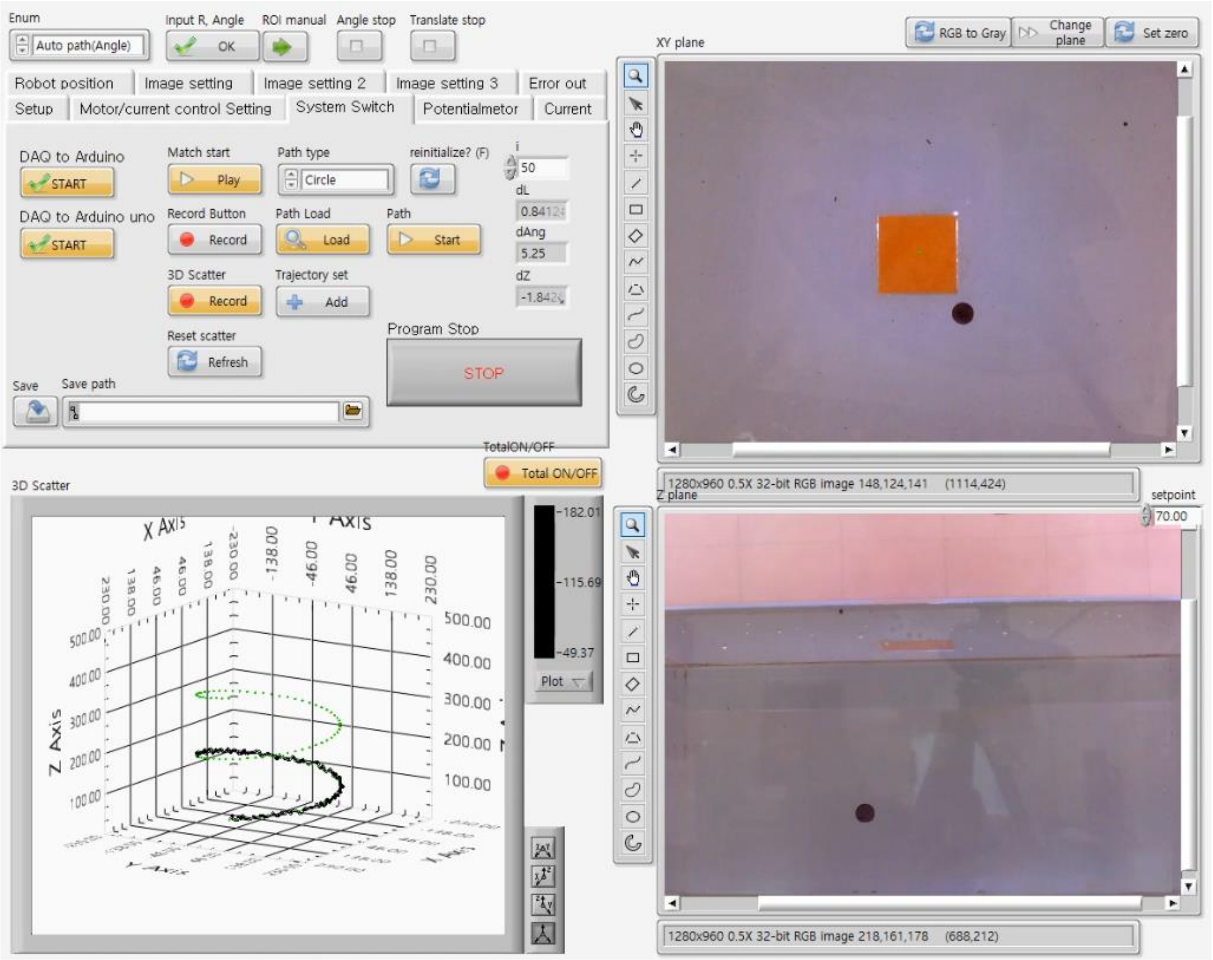

Figure S2. Graphic User Interface of the control system.

The Graphic User Interface (GUI) developed for the locomotion control of mag-bots is shown in **Figure S2**. The GUI is divided in four main sections, where the right upper section displays the XY plane, whereas the right lower section displays the ZX plane. The left lower displays the input 2D or 2D trajectory in green, and the measured position of the mag-bot in black. The remaining left upper section contains different menus where the user can select the among the different control modes (TL, CL, OL, CL), the current in the coil, adjust the parameters for image tracking and monitor the position of the linear actuators.

A diagram of a parallel robot is shown in **Figure S3**. The linear actuator length vector $\mathbf{l}_i$ is given by

$$\mathbf{l}_i = \mathbf{t} + \mathbf{R}\mathbf{p} - \mathbf{b}, \quad \text{(S1)}$$

where $\mathbf{t}$ is the vector pointing from the origin of the base (O_B) to the origin of the coil (O_C), $\mathbf{b}_i$ is the vector pointing from O_B to the base of the linear actuator B_i, $\mathbf{p}_i$ is the vector pointing from O_C to the other end of the linear actuator A_i, and $\mathbf{R}$ is the 2D rotation matrix including the yaw (α) and pitch (β) angles. $\mathbf{R}$ is expressed as

$$\mathbf{R} = \begin{bmatrix} \cos\alpha\cos\beta & -\sin\alpha & \cos\alpha\sin\beta \\ \sin\alpha\cos\beta & \cos\alpha & \sin\alpha\sin\beta \\ -\sin\beta & 0 & \cos\beta \end{bmatrix}. \quad \text{(S2)}$$

Although the platform can also perform a rotation in the Z-axis, such rotation has no effect on the mag-bot. Therefore, it is omitted in the control equations. Moreover, α and β are defined by ϕ and the angular coordinate θ as follows:

$$\alpha = \phi \sin\theta, \quad \text{(S3)}$$

$$\beta = \phi \cos\theta. \quad \text{(S4)}$$

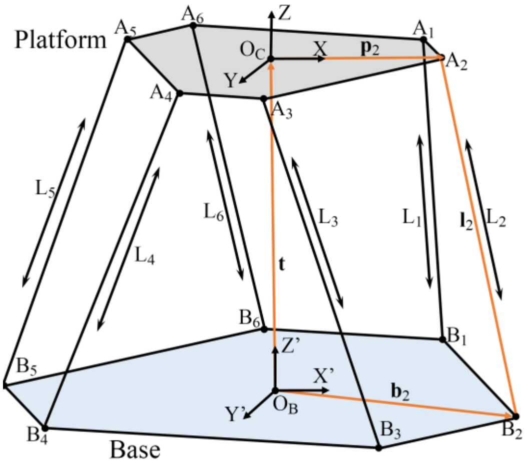

Figure S3. Parallel robot model.

Section S2. Working Range for MB1

To calculate the working range for a given microrobot, the required magnetic gradient $\mathbf{G}_z$ that needs to be applied to fulfill Equation 4 has to be calculated. The parameters used in our calculations for locomotion of MB1 are summarized in **Table S1**.

Table S1. Parameters for force calculation

Parameter	Value
V_r	0.286×10^{-6} m^3
m_r	0.717×10^{-3} kg
m	0.0457 A·m^{-2}
ρ_f	1192.15 kg/cm3
μ_f	45.38 mPa·s
F_b	3.3 mN
F_g	7.0 mN
F_d	4.27 µN

According to the forces shown in Table S1, the applied $\mathbf{G}_z$ needs to be 80.6 µT·mm^{-1} < $\mathbf{G}_z$ <154 µT·mm^{-1}. **Figure S4** shows the distribution of $\mathbf{G}_z$ along the axis of the coil for different values of I_c, delimiting with black dashed lines the range where MB1 can be levitated for its

control. It shows that for an I_c = 30 A, the maximum distance between the center of the coil and MB1 is about 275 mm.

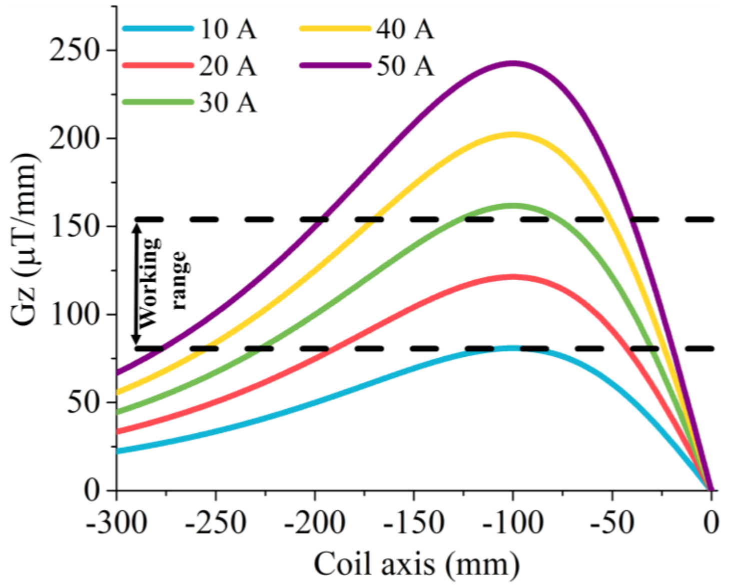

Figure S4. G$_z$ distribution along the coil axis and working range for MB_1

Section S3. 2D Locomotion Controllers

To control the locomotion of the mag-bot in the XY plane, the desired set of positions for the TP (towards which the mag-bot will be dragged) is input in cylindrical coordinates, where r_{ref} and θ_{ref} are the desired radial distance and angular coordinate, respectively. For locomotion control that uses a constant tilting of the coil ϕ = 0º, O_C is displaced to match the desired position of the TP for locomotion control using the translation motion of the coil. Because the distribution **G**r does not change with respect to the coil when the coil was translated, we considered that the origin of the TP (O_{TP}) is also translated. Therefore,

$$\begin{bmatrix} r\cos\theta \\ r\sin\theta \end{bmatrix} = O_c = O_{TP}\,. \tag{S5}$$

For the tilting control, the position of the TP with respect O_{TP} is

$$\begin{bmatrix} r\cos\theta \\ r\sin\theta \end{bmatrix} = f(r)\begin{bmatrix} \cos\theta \\ \sin\theta \end{bmatrix}, \tag{S6}$$

where $f(\phi)$ is a function that relates the tilting angle of the coil and the magnitude of the displacement of the TP, which can be obtained through curve fitting of the experimental data or calculations using Biot–Savart law.

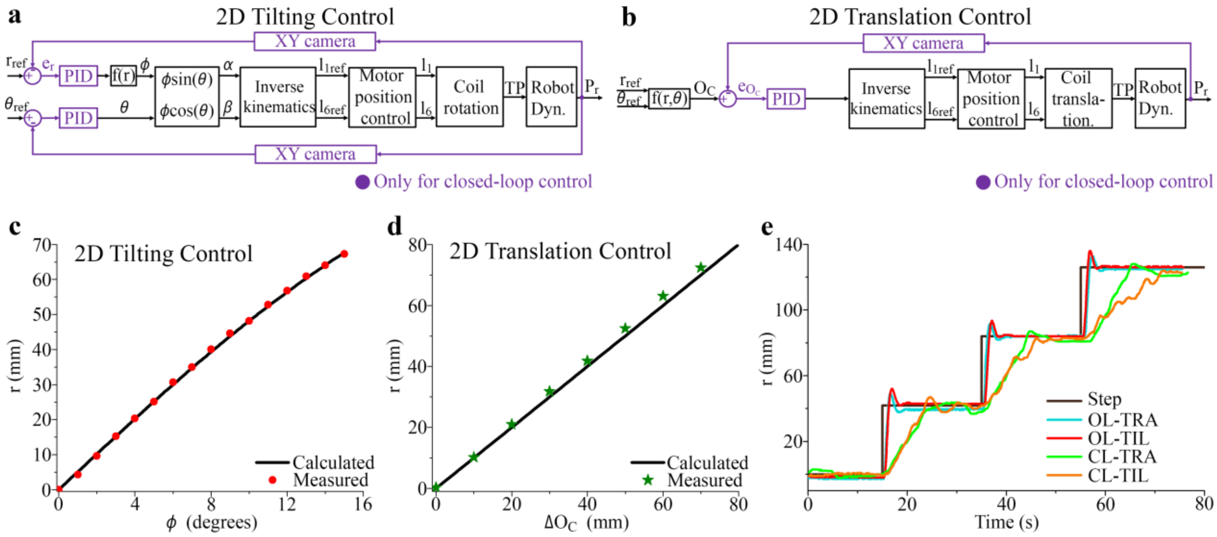

Figure S5. Block diagrams for the 2D open-loop (OL) and closed-loop (CL) automatic locomotion control of a mag-bot using the tilting (TL) (a) and translation (TRA) (b) motion control of the coil. Calculated and measured position of the mag-bot according to the coil tilting angle (c) and the coil translation (d). e) Step response of the four 2D control systems.

Figure S5a and S5b show the block diagram for the feedbackless (open-loop (OL)) and closed-loop (CL) locomotion control of a mag-bot using tilting (TIL) or translation (TRA) control of the coil. The blocks and variables in black apply to both OL and CL control systems, whereas the blocks and variables in purple apply only to CL control systems. For the

OL-TIL control, r_{ref} and θ_{ref} are fed to the control system, which uses Equation S3, S4, and S6, and calculates the required rotations α and β that the parallel robot needs to perform. Then, using Equation S1 and S2, the required length for each linear actuator is controlled, resulting in the rotation of the coil and the displacement of the TP, which drag the mag-bot along with it. The new position of the O_C is calculated using the control parameters r_{ref} and θ_{ref} and Equation 5 when the OL-TRA control is used. Subsequently, the translation motion that the parallel robot must perform and the length of the linear actuators are calculated. The system moves the coil to the desired position, resulting in TP and mag-bot displacements. For CL-TIL and CL-TRA, the position of the mag-bot (P_r) is fed back to the system through the XY camera, and compensation is performed using a PID controller.

Figure S5c and S5d show the respective calculated values using Biot–Savart law and measured values for the position of the mag-bot according to the tilting and translation of the coil performed by the parallel robot for a separation distance between O_C and the XY plane of 190 mm. As observed, both the measured and calculated values were similar. Furthermore, the relationship between ϕ and r is obtained in Equation S7 by curve fitting the data in Figure S5c.

$$\phi = 0.0007489r^2 + 0.1662r + 0.224 \quad \text{(S7)}$$

The curve fitted relation between ΔO_C and r is

$$\Delta O_c = 0.9671r - 0.3576\,. \quad \text{(S8)}$$

The step response for an increase in r of 40 mm for each of the four 2D control systems is shown in Figure S5e. The response of both OL systems is the fastest but has an overshoot produced by the momentum of the mag-bot as it reaches the TP, after which the mag-bot experiences a magnetic force in the opposite direction, pushing it back to the TP. The overshoot in OL systems can be reduced by decreasing the step size because the momentum of the mag-bot will be smaller when reaching the TP. In the case of CL control systems, the step response is slower and slightly oscillatory, but the overshoot is also smaller.

Section S4. 3D Locomotion Controllers

The block diagrams for the 3D locomotion of a mag-bot using tilting and translation controls are shown in **Figures S6**a and S6b, respectively. In both cases, the control systems are the same as their 2D versions, but with the addition of levitation control, as shown in red. To control the vertical position of the mag-bot, the desired vertical position Z_{ref} is fed to the system and compared with the measured position of the robot (performed by the XZ camera) to increase or decrease the value of I_C using a PID controller.

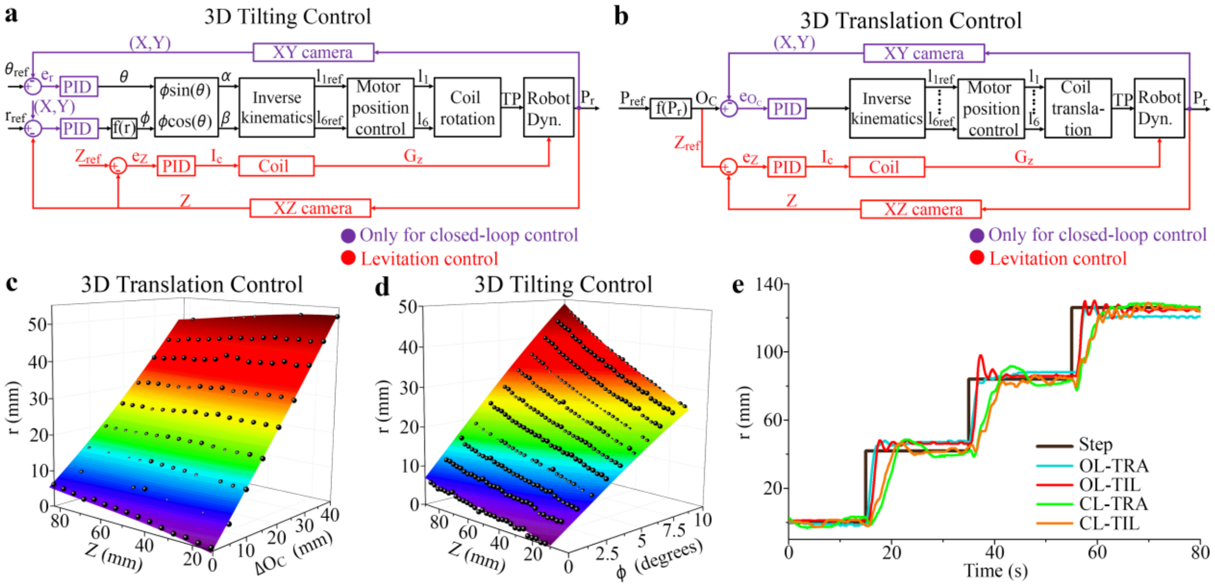

Figure S6. Block diagrams for the 2D open-loop (OL) and 2D closed-loop (CL) automatic locomotion control of a mag-bot using the tilting (TL) (a) and translation(TRA) (b) motion control of the coil in addition to levitation control for 3D locomotion control of a mag-bot. Fitted and measured position of the mag-bot according to the coil translation (c) and the coil tilting angle (d). e) Step response of the four 3D control systems.

As previously discussed in Section 2.1, the relative position of the TP remains constant for all planes perpendicular to the Z-axis below d_c when O_C changes, whereas the position of the TP changes with the vertical position of the mag-bot when ϕ changes. Therefore, the position of the mag-bot was measured and curve-fitted at the ZX plane of the working space according to the displacement of O_C (ΔO_C) and ϕ to obtain a mathematical expression that relates r to Z and ϕ and to compensate for the physical imperfections of the system, as observed in Figure S6c and S6d, respectively. The curve fitting of the data in Figure S6c provides the following relation:

$$\begin{aligned}\phi &= 0.4141 + 0.09834r - 0.01399z + 1.258\times10^{-4}r^2 - 1.82\times10^{-4}rz + 5.004\times10^{-5}z^2 \\ &-2.66\times10^{-7}r^2z + 1.498\times10^{-7}rz^2 - 7.151\times10^{-8}z^3\end{aligned}, \quad \text{(S9)}$$

and the curve fitted relation between $\Delta O_{C,}$ Z, and r is

$$\begin{aligned}\Delta O_c &= 2.232 + 0.1828r - 0.03628z - 3.543\times10^{-6}r^2 + 2.322\times10^{-4}rz + 8.985\times10^{-5}z^2 \\ &-5.63\times10^{-7}r^2z + 9.521\times10^{-8}rz^2 - 1.339\times10^{-7}z^3\end{aligned}. \quad \text{(S10)}$$

The step response for an input of 40 mm for each of the four 3D controls is shown in Figure S6e. As with the 2D systems, the step response for the OL systems was the fastest and had some overshoot, whereas the CL systems showed a slower response, slight oscillation, and slightly higher precision.

Section S5. G_z Distribution with respect the Radial Distance

Figure S7 shows the distribution of $\mathbf{G_z}$ with respect to the radial distance from the coil axis at different separation distances from the coil. As observed, the vertical force that a mag-bot experiences depends on both Z (the vertical distance between the coil and the mag-bot) and *r*, existing two maxima for Z= 140 and 180 mm. However, as the distance increased, only one maximum was located at the coil axis.

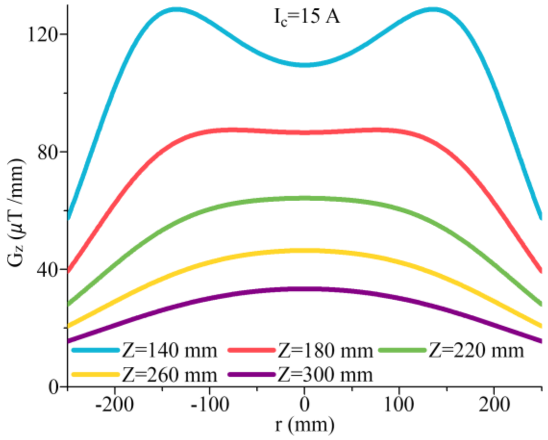

Figure S7. G_z with respect r for different separation distances from the coil axis, with I_c=15A.

Supporting Videos

Video S1: 3D Locomotion using tilting control & current control.

Video S2: 3D Locomotion using translation control & current control.

Video S3: 2D feedbackless locomotion control using hybrid mode.

Video S4: Selective control of two magnetic millirobots.

Video S5: Locomotion of a mag-bot and retrieval of MNP in a complex biological fluid.
